# A Sustainable Recycling Alternative: Electrospun PET-Membranes for Air Nanofiltration

**DOI:** 10.3390/polym13071166

**Published:** 2021-04-05

**Authors:** Daniela P. F. Bonfim, Fabiana G. S. Cruz, Rosario E. S. Bretas, Vádila G. Guerra, Mônica Lopes Aguiar

**Affiliations:** 1 Departamento de Engenharia Química, Universidade Federal de São Carlos–UFSCar, Rodovia Washington, Luiz, km 235–SP 310, São Carlos 13565-905, Brazil; bonfimdaniela22@gmail.com (D.P.F.B.); fabigscruz@gmail.com (F.G.S.C.); vadila@ufscar.br (V.G.G.); 2 Departamento de Engenharia de Materiais, Universidade Federal de São Carlos–UFSCar, Rodovia Washington Luiz, km 235–SP 310, São Carlos 13565-905, Brazil; bretas@ufscar.br

**Keywords:** microfiber, filter media, electrospinning, nanofiltration, nanotechnology, recycling materials

## Abstract

Currently, the inappropriate disposal of plastic materials, such as polyethylene terephthalate (PET) wastes, is a major environmental problem since it can cause serious damage to the environment and contribute to the proliferation of pathogenic microorganisms. To reduce this accumulation, PET-type bottles have been recycled, and also explored in other applications such as the development of membranes. Thus, this research aims to develop electrospun microfiber membranes from PET wastes and evaluate their use as an air filter media. The solution concentrations varied from 20 to 12% wt% of PET wastes, which caused a reduction of the average fiber diameter by 60% (from 3.25 µm to 1.27 µm). The electrospun filter membranes showed high mechanical resistance (4 MPa), adequate permeability (4.4 × 10^−8^ m^2^), high porosity (96%), and provided a high collection efficiency (about 100%) and low-pressure drop (212 Pa, whose face velocity was 4.8 cm/s) for the removal of viable aerosol nanoparticles. It can include bacteria, fungi, and also viruses, mainly SARS-CoV-2 (about 100 nm). Therefore, the developed electrospun membranes can be applied as indoor air filters, where extremely clean air is needed (e.g., hospitals, clean zones of pharmaceutical and food industry, aircraft, among others).

## 1. Introduction

Since the 1960s, polymers have been increasingly replacing conventional materials like metal and wood [1]. Not only because of their lower cost but also due to the continued development of their functionality, which allows its use in various applications, from packaging to civil construction [2]. Recycling of waste plastics has received significant worldwide attention because it can reduce production costs (regarding the use of fossil fuels for production) and minimize the overall impact on the environment [3,4,5]. 

Polyethylene terephthalate (PET) is a highly used polymer, mainly in the production of bottles for beverages, whose global production went from 300 billion in 2000 to 480 billion in 2016 and its consumption is still increasing, so the expectation is that in 2021 it may reach 583 billion bottles [6]. As a result, the waste generated by PET bottles is of great concern due to the improper disposal of post-consumer packaging. This occurs, for example, when PET waste blocks the drains of water bodies, leading to overflows and sometimes flooding, as well as accumulation in open dumps [7].

Using recycled PET is a viable option to mitigate PET waste since it can be recycled multiple times into a wide range of products. Although there are many advantages to using recycled PET to replace virgin PET, not all demand for PET can be met through recycling [6]. Bottle-grade PET exists as a semi-crystalline thermoplastic with high impact and tensile strength, chemical resistance, and reasonable thermal stability [2], which enables its use in various applications, such as in the development of nanofibers and composites [8,9,10]. 

There are essentially three different destinations for plastic waste, the first of them being recycling or reprocessing into a secondary material. Second, plastics can be destroyed thermally. Finally, plastics can be discarded and even contained in a managed system [1,3,11]. However, regarding the reprocessing, using recycling material also has its challenges (e.g., unstable quality, impurities) since the pure polymer is mixed with additives to enhance the properties of the material [1]. In this work, to minimize the effect of these additives and also the recycling process that causes a harmful change in the properties of the material, only bottles with the same volume from the same manufacturer were utilized. Moreover, only the central part of the bottles was used. The reuse of waste as a source of raw material for making filter media is a sustainable and energy-friendly alternative since it favors the use of renewable energy sources and has been widely reported in the literature [8,12,13,14,15], as the manufacture of a functionalized magnetic fullerene (FMFN) nanocomposite associated with sustainable residues from PET bottles [16], and in civil construction, with recycled PET fibers being used to reinforce the mechanical properties of construction materials [11,17,18,19].

Air pollution is another major problem of the 21st century. According to the World Health Organization, 7 million people every year die due to environmental pollution, especially due to the fog caused by particulate matter [20]. Exposure to those particles has been strongly associated with adverse health effects including physiological impairments, psychological impacts, increase in expenses due to the loss of productivity at work, besides the increase in healthcare expenditure [21,22,23,24]. Furthermore, airborne microorganisms as viruses, bacteria, and fungi (and their fragments), are also classified as particulate material, or more specifically, bioaerosols, that vary in size, from submicroscopic particles (<0.01 μm) to particles larger than 100 μm, which are easily carried by the wind and can float for a long time in the atmosphere [25]. If inhaled or adhered to by humans, they become a dangerous group of etiological agents for human respiratory and infectious diseases, like Influenza virus, Rhinovirus, Mycobacterium tuberculosis, and the SARS-CoV-2, which causes the disease that is currently a major public health problem, COVID-19 [26,27]. 

Generally, the control of particulate matter and airborne microorganisms, mainly in indoor environments, is done through traditional filtration that is the most widely used approach [28,29,30]. Currently, several types of research point to the effectiveness of membranes applied to air filtration since electrospun filter media can achieve collection efficiency for particles smaller than 2.5 µm (very close to 100%), low-pressure drop and permeability below the values obtained for HEPA filters [31,32,33]. An example, polyamide electrospun nanofibers reached efficiency values close to 98.79% and 96.79% [29,34]. About the current scenario, the new coronavirus has a size of 60–140 nm with an average size in nanoaerosols of 100 nm, which makes the use of membranes with 100% collection efficiency for this range of particle diameters very attractive due to the growing demand for occupational safety and public health in this pandemic situation [35]. 

Nonwoven fabrics of PET could here play a momentous role in dust filtration because of their porous structure and low cost of manufacturing in combination with their unique mechanical properties such as high mechanical resistance [8,11,14,17]. This characteristic was a differential in this work since the high mechanical resistance of the developed fibers maintained the structural stability during the filtration tests.

Electrospinning techniques are a viable alternative and already widely used to produce micro and nanofibers, which differs from other fiber production processes in the ability to control the diameter, morphology, orientation, and structure of the fiber and their chemical composition [13,36,37]. However, one of the challenges to produce these filter media is the adjustment and control of several parameters which influence fiber production, (explored in this work) [38,39,40,41,42]. Thus, the utilization of this technique to produce filter media from post-consumer PET bottles is a very promising and ecologically sustainable alternative because it highly contributes to the reduction of environmental pollution [6,43].

Thereby, the objective of this study was to produce, by electrospinning, resistant filter media with microfibers obtained from PET bottles waste for the highly efficient removal of nanoparticles present in the air. Besides that, the influence of electrospinning parameters on the structural and morphological properties of the filter media was systematically investigated. Due to the excellent mechanical properties of electrically spun PET waste membranes, they can expand their potential application to environmental remediation as a filter media as they can be used as individual protection (masks), cleanroom (biomedical, electronics, pharmaceutical, automotive), and indoor air purification (airline cabin).

## 2. Materials and Methods

### 2.1. Materials

To produce the filter media, the following materials were used: trifluoroacetic acid (TFA—Neon, Suzano, Brazil), dichloromethane (DCM—Synth, Diadema, Brazil), 10 mL plastic syringes (BD Plastipak, Curitiba, Brazil), needles with a diameter of 0.7 mm (BD Precision Glide, Curitiba, Brazil) and sodium chloride (NaCl—99%, Sigma Aldrich, St. Louis, MO, USA) for the nanoparticles generation. 

### 2.2. Preparation of PET Microfibers

The fibers were produced by electrospinning using 500 mL post-consumer PET bottles as the polymer. The bottles were first washed with distilled water, sprayed with ethanol in excess, and dried. Then the central parts of these bottles were crushed into small pieces that were added, in the proportion of 12 and 20% by weight, in a solvent mixture composed of trifluoroacetic acid (TFA) and dichloromethane (DCM) in the proportion of 70/30%. The obtained solutions were stirred (750 rpm) for 3 h at room temperature (25 °C), inside a hood, until forming a homogeneous solution, whose viscosity decreased from 1286.7 cP to 221.7 cP. The microfibers were prepared using a combination of the best conditions reported in the literature for the production of polymeric fibers by electrospinning [8,10,14,40] and were named PET20_0.8, PET12_0.8, and PET12_1.0, whose variations in operating parameters are shown in Table 1. A syringe pump (KDS 100, KD Scientific, Holliston, MA, USA) was used to feed the solution at two flow rates. A high voltage power supply (T1CP 300 304n-iSeg, Radeberg, Germany) was used to supply 20 kV of energy. The distance between the needle and the collector was maintained at 10 cm and the speed of the collector at 357 rpm. Viscosity was measured using a Brookfield spindle SC4-18 viscometer (Brookfield LV-DVIII, Brookfield Engineering Laboratories Inc., Middleboro, MA, USA).

### 2.3. Characterization of Fibers

The morphology of membranes was examined by Scanning Electron Microscopy (SEM) using a microscope from Hitachi S4800, (Tokyo, Japan), and the size distribution of the fibers and thickness was obtained by an image analysis software (Image J1.29X) as described by Bortolassi et al. (2019) and by Salussoglia et al. (2020) [31,44]. The fibers’ layers were characterized by analysis of images with 500× and 4000× of magnification. Two samples from each filter media were analyzed, with five images obtained for each sample (totaling 10 images per filter media). The tensile strength of the electrospun fibers was analyzed using a dynamic mechanical analyzer from TA Instruments (Model Q800, New Castle, DE, USA) in this equipment, the samples have dimensions between 5.40 to 5.50 mm in length and 6.80 to 7.00 mm in width. Material elongation was performed at a speed of 700 µm/min from 0 to 10,000 µm at room temperature (25 °C) in air. The permeability is an important transport property, which is connected to the geometric structure of porous fibrous materials because can influence the flow of fluids through these materials [45], so the arrangement of the fibers can be challenging or favorable to the airflow passing through the filter. Thus, permeability is the estimation of the easiness of the flow of fluids in fibrous materials. The permeability constant (K_1_) was obtained using Darcy’s Equation (Equation (1)) (tested in duplicates) and the volumetric flow rate was varied from 100 to 2000 mL/min for a filtration area of 5.2 cm^2^:

∆P/L = (μ/K_1_) × (v_s_) (1)

where μ is the gas viscosity, L is the thickness of the filter media, v_s_ is the filtration superficial velocity and ∆P is the pressure drop; this last parameter was measured using a digital manometer (VelociCalc Model 3A-181WP09, TSI, Shoreview, MN, USA) connected to the filtration apparatus, as described by Bortolassi et al. [31,32]. Thus, the permeability is explicitly related to the microstructural parameters of fibrous porous media [45,46]. The empirical porosity (ε) of the filter media was calculated as proposed by Ergun’s Equation in 1952, as presented follow (Equation (2)):

∆P/L = (150(1 − ε)^2^μv_s_)/(ε^3^·d_f_^2^) + (1.75(1 − ε) ρ_g_ v_s_^2^)/(ε^3^d_f_) (2)

where ρ_g_ is the gas density and d_f_ is the average diameter of the fiber. The porosity was determined theoretically to evaluate the void fraction between the fibers.

### 2.4. Filtration Performance 

The collection efficiency (Equation (3)) for particles was also determined, with particle diameters ranging from 7 to 300 nm at a filtration velocity of 4.8 cm/s, a flow rate of 1500 mL/min, and a filtration area of 5.2 cm^2^ (kept constant). The nanoparticle concentration upstream (C_0_) and downstream (C_1_) of the filter media was calculated using a differential mobility analyzer and particle counter (Model 3776, TSI, Shoreview, MN, USA), as described by Bortolassi et al. (2019) [31] from the following equation:

E = (C_0_ − C_1_)/C_0_ (3)

According to the classical filtration theory, there are mainly five mechanisms to retain particles during filtration: interception, inertial impact, Brownian diffusion, gravitational effect, and electrostatic effect. The predominance of one or more mechanisms is related to the size of the particle to be filtered, the gas flow rate, the characteristics of the fluid, permeability, porosity, and the fibers’ diameter that constitutes the filter media as well as its distribution, the latter being amenable to control by the electrospinning technique [47].

## 3. Results

### 3.1. Characterization of Microfibers

The morphological characteristics such as thickness, average filter media diameter, tensile strength, and porosity are presented in Table 2.

**Figure 1:**
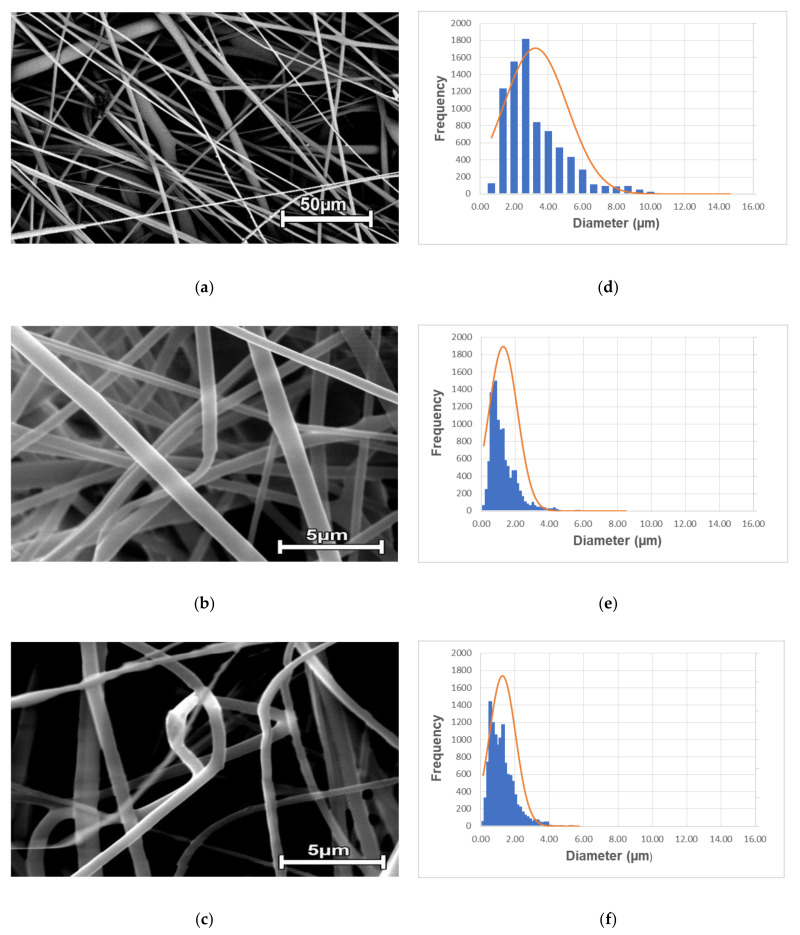
SEM images of electrospun recycled PET fibers: PET20_0.8 (**a**); PET12_0.8 (**b**) and PET12_1.0 (**c**), with their respective fiber size distribution (**d**–**f**).

The different conditions of the PET bottles waste solutions and the electrospinning technique produced microfiber filter media that can be applied in air filtration. The influence between the polymer concentration and the microfiber morphology was investigated: the reduction in the value of the average fiber diameter occurred when the concentration of recycled PET decreased from 20 to 12% in weight, as shown in Figure 1.

In this figure images of the microfibers obtained by scanning electron microscopy (SEM) and their respective fiber diameter distribution are shown, which were determined using the methodologies reported in the literature [31,44]. The enlargement of Figure 1a differs from the others to facilitate visualization of the fiber size distribution. By decreasing the concentration of post-consumer PET bottles, the solution’s viscosity decreased from 1286.7 cP to 221.7 cP, as expected. In order to obtain a filtering media with recycled PET microfibers, with ideal characteristics to be applied in air filtration, several parameters were varied; this article presents the best results. Among the parameters investigated, the concentration of recycled PET was the one that influenced the most the average fiber diameter, a result also obtained in other studies [36,48,49]. This occurs because the polymer concentration is associated with the solution’s viscosity and consequently influences the electrospinning jet [47,50,51], which depending on its elongation, can form thinner or thicker fibers. As described by Haider et al. (2018) [52], the electrospinning process depends on the uniaxial elongation of a charged jet.

Thus, more viscous solutions, like PET20_0.8, have the most tangled polymer chains and, consequently, less mobility in the solution due to the high viscoelastic forces. This makes the stretching of the jet during the process more difficult, resulting in larger fiber diameters as observed for the sample PET20_0.8 (see Table 2). However, solutions with very low concentrations do not resist the deformation of the fiber under the action of the applied electric field before reaching the collector, causing thinner fibers [49,51,53]. There is an ideal viscosity in which the stable and continuous jet allows the formation of fibers without defects, consequently, the ideal concentration must then be determined based on the morphology of the fibers to be produced [52]. The goal of this work was to develop filter media with fine fibers without structural defects. According to Matulevicius et al. (2014) and Nezarati et al. (2013), the polymer viscosity significantly changes the fiber diameter, that is, the lower the viscosity, the smaller the diameter of the fiber [40,54]. Considering that, when a solid polymer is dissolved in a solvent, the solution viscosity is directly proportional to its concentration. To obtain filter media with fibers of smaller diameters, tests were carried out using lower polymers concentrations, from 20 to 12% in weight. Thus, it was possible to observe that the smallest fiber diameters were found for the samples of PET12_0.8 and PET12_1.0.

These values obtained for the diameters of the PET bottles waste microfibers were close to the values of the microfibers of commercial filter media, such as those of glass fibers [55] and those of cellulose fibers [56], which presented values of average diameters close to 1 µm, whereas those of polypropylene fibers were around 2.2 µm [55]. Other researchers obtained average diameter values for recycled PET microfibers, used in the filtration of wastewater, of approximately 1.04 µm [10].

It should be noted that the sample PET20_0.8 presented a higher average diameter and a greater size distribution, between 0 and 10 µm, while the other samples presented close average diameters and smaller diameter distribution range. Therefore, the fiber PET20_0.8 can be characterized as more heterogeneous than both PET12_0.8 and PET12_1.0 samples. However, samples PET12_0.8 and PET12_1.0 showed a higher frequency of fibers with diameters less than 2 µm.

The filter thickness can be controlled by the time of collection of the fiber and the distance between the needle and the collector [34,40,57,58,59]. In this sense, the thickness must be controlled so as not to compromise the air filtration process, that is, the collection efficiency of the nanoparticles and the filter pressure drop [58]. In the present study, it was observed that the produced filter media had slightly different thicknesses (see in Table 2); for all samples, the collection time was 6 h and the distance from the needle to the collector was 10 cm. A slight increase in thickness was observed for samples with the same concentration (PET12_0.8 and PET12_1.0). It can be inferred that this increase was caused by the increase in the feed rate, from 0.8 to 1.0 mL/h. These results also were observed by Strain et al. (2015) whose flow rate was three times high (at a given concentration) and had only a marginal effect on the thickness of the fibers [14]. The solution flow rate into the needle influences the droplet size and, consequently, affects the fiber morphology. Therefore, different morphologies can be obtained with the change in feeding rate at a given electric field [51] since the flow determines the amount of solution available for electrospinning [60]. If the flow velocity is too high, the jet cannot be fully stretched by the electrostatic field, so there will be a corresponding increase in droplet size, generating fibers with larger diameters, while the opposite is also true to obtain smaller diameters [37]. In this work, the flow reduction did not result in a significant decrease in the average diameter, as expected, since concentration and viscosity (which were the same) are the parameters that exert the most significant influence in diameter, as cited before. However, it is possible to observe a small difference between the diameter distribution. Although both have microfibers diameters in the range of 0 to 4 µm, the frequency of smaller diameters is higher in sample PET12_0.8, with the Gaussian curve exceeding the 1800 value (see Figure 1e). Probably this higher frequency of smaller diameters resulted in the smaller thickness of this sample because the collection time and the concentration were the same. The PET12_1.0 sample, on the other hand, showed a higher frequency of larger diameters (see Figure 1f), which may explain its slight increase in thickness. This can be interpreted as a consequence of the mats with thick fibers having larger pores [14]. Such an increase in the average pore diameter resulted in an increase in porosity (from 92 to 96%) and, consequently, in permeability constant (from 1.07 × 10^−8^ to 4.4 × 10^−8^ m^2^) for samples PET12_0.8 and PET12_1.0, respectively. This occurs, probably because the large porosity leads to an increase in fluids flow rate and consequently increases the permeability [46] since the permeability is explicitly related to the microstructural parameters of filter media.

The porosity of the samples was 94% for PET20_0.8, 92% for PET12_0.8, and 96% for PET12_1.0. For samples with the same flow rate, which explains the greater porosity of PET20_0.8 concerning PET12_08, it is the largest diameter of the fibers [14] which influences more. The influence of these characteristics on filtration performance will be discussed later. The porosity of a filter media may change the collection efficiency for the particle size range studied, however, it is not capable of interfering in the behavior of the theoretical curve, since it will follow the trend of the classical filtration theory [36].

**Figure 2:**
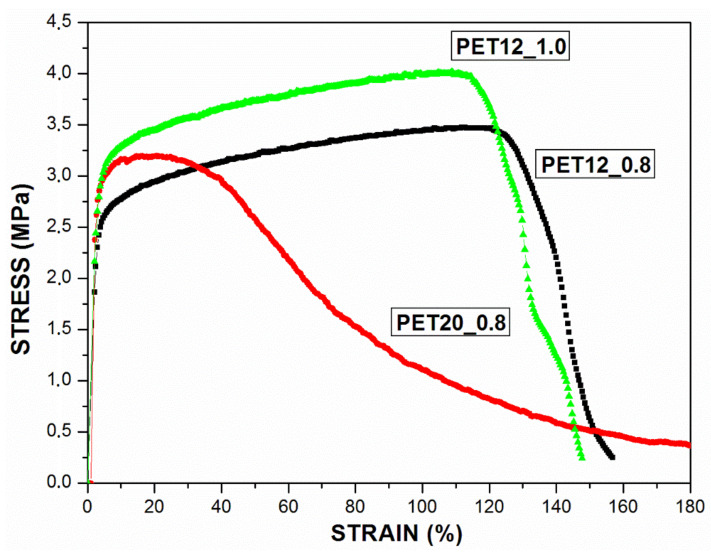
Tensile stress–strain curves for the PET20_0.8, PET12_0.8 and PET12_1.0 samples.

The breaking mechanism of the fibers’ tensile fracture under external stress was analyzed, as shown in Figure 2. The fibers exhibited mechanical properties in the horizontal direction favorable to filtration tests. The membranes exhibited linear elastic behavior in the first region under a stress load until reaching yield point. As can be seen in the initial region of the curves, Young’s modulus of the three samples is remarkably similar, meaning that they present similar behavior of elastic deformation.

The tensile strength can be determined by the maximum point of the curve and translates into how much the material can withstand the stress being applied. The tensile strength reached 3.2 MPa, 3.5 MPa, and 4 MPa, for samples PET20_0.8, PET12_0.8, PET12_1.0, respectively.

These values are close to those reported by other authors for use in filtration operations where fibers were produced by electrospinning of PAN polymeric solutions, which had a resistance of 3.8 MPa [61], PVC in the order of 1.0 MPa [60], and pure PLA of 2 MPa [62].

It should be noted that the behaviors of PET12_0.8 and PET12_1.0 were very similar, with both presenting a remarkably high elongation until reaching the tensile strength limit, as can be observed in the tensile stress–strain curves. Such samples deform plastically as a whole, which justifies the higher tensile strength. In comparison, the behavior of sample PET20_0.8 is different, considering it reached the limit of tensile strength without presenting a high elongation. However, it is possible to observe from the stress–strain curve that, after reaching this limit, the fiber does not break, so it can be inferred that initially a stress concentration zone was formed and it deforms plastically from that point onwards. Toughness is defined as the amount of energy that the sample absorbs before breaking (area under the stress–strain curves); therefore, it can be concluded that samples PET12_0.8 and PET12_1.0 were tougher than sample PET20_0.8., probably due to their lower fiber diameters.

As concluded before, as the fiber diameter decreased, an increase in strength was observed. This could be explained by a higher degree of molecular alignment by the electrical field when thinner fibers were produced, due to the increased fiber stretching, as it has been reported in the literature, in which recent experimental studies have demonstrated improvements in modulus and strength of electrospun polymer nanofibers with reduction of their diameter [63].

Another parameter that influences the diameter of the fibers is the distance between the needle tip and the collector as well as the electrical voltage applied. These two variables are related, as both determine the intensity of the electric field applied in the process. According to the literature, the diameter of the fibers varies with the length of the jet, meaning that if there was a reduction of the working distance, consequently, there would be a reduction in diameter [36,47]. Concerning the increase of the applied electric voltage, while keeping the tip to collector distance constant, it is possible to verify a reduction in diameter since the voltage presents as a variable that has a critical value, which is related to the tip to collector distance [37,47,64]. Thus, the working distance (10 cm) and the applied voltage (20 kV) were previously determined and kept constant in all experiments reported in this paper so that the influence of other variables could be assessed separately.

### 3.2. Performance Filtration

Table 3 shows the characteristics related to the filtration performance of the microfibers studied in this work. Properties such as permeability constant, pressure drop, and collection efficiency (from 3 to 300 nm nanoparticles) will be analyzed in sequence.

Analyzing the results obtained in this study (Table 3), the highest K_1_ value was obtained for sample PET20_0.8, which has a more open structure (Figure 1), resulting in a lower pressure drop value of 13.5 Pa, while the lowest K_1_ value was obtained for the PET12_0.8 sample, with the more closed structure. In this sense, the highest pressure drop found for sample PET12_0.8, equals 212.5 Pa, was due to the greater resistance offered to the flow passage compared to sample PET12_1.0, with a pressure drop value of 64.8 Pa. Thus, due to its more closed structure, the greatest collection efficiency for the nanoparticles (98.4%) was found for the membrane with the greatest pressure drop (PET12_0.8). Comparing the permeability values found for the filter media produced in this study with the values reported in the literature, the values obtained here were higher. Yun et al. (2007) [59] reported K_1_ values in the order of 10^−12^ m^2^, for a HEPA filter. Bortolassi et al. (2019) [31] obtained values in the order of 10^−13^ m^2^, for polyacrylonitrile nanofilters. Thus, it is possible to conclude that the filters produced in this work have a more open structure, with a lower pressure drop compared to the HEPA filters reported by Yun et al. [59].

The nanoparticle filtration is quite complex and there is no specific value for the variables related to the structure to obtain the best filter media performance, but a combination of parameters like permeability constant, porosity, pressure drop, thickness, among others are determinant. The complex transport behavior in fibrous porous media, however, makes it difficult to determine its geometric structure [65]. In recent years, fractal theory [66] had been widely used to develop mathematical models. These models are based on the fractal distribution of pore size in fibrous porous media, and thus can be expressed as a function of structural parameters of fibrous porous media, including porosity, micro-pore size, fiber diameter, tortuosity fractal dimension, and area fractal dimension of pores [46]. According to the fractal model, the physical mechanisms of fluids transport through fibrous porous media are better elucidated, as reported in the literature [45,67]. Xiao et al. (2019) proposed a fractal solution to investigate the permeability and the Kozeny–Carman (KC) constant of fibrous porous media that presented an agreement with data obtained in the laboratory, analytical solution, and numerical simulation in the literature. The results demonstrated, among others, that an increase in the porosity and the fiber diameter yields an increase in the absolute permeability [46]. Such a result is consistent with this study. 

Therefore, the best configuration of these parameters allows the correct performance of the collection mechanisms (diffusion, inertial impaction, interception, gravitational, electrostatic) between the fibers and the nanoparticles [36]. The filtration efficiency depends on the performance of each of these collection mechanisms [21], with the diffusion mechanism being the predominant one for particle diameter range in this study [36]. Filtration tests were made with nanoparticles whose maximum diameter was equal to 300 nm, that is, much smaller than the PM2.5 (2500 nm). This means that these filters presented high collection efficiency for nanometric particles. Based on this, it can be inferred that filter PET12_1.0 could also achieve collection efficiency close to 98.4% for PM2.5, the main air pollutant problem.

It was also observed that the reduction of the fiber diameter led to the increase of pressure drop, as can be seen in Table 3. Sample PET12_0.8 displayed a higher pressure drop when compared to the other samples and, consequently, the filtration efficiency increased. A greater variation of pressure drop suggests that the fibers are more intertwined and therefore offer greater resistance to airflow as it passes through the filter media, as described before [36,40]. Thus, airborne particles were more easily collected, which contributed to the increase in collection efficiency. 

**Figure 3:**
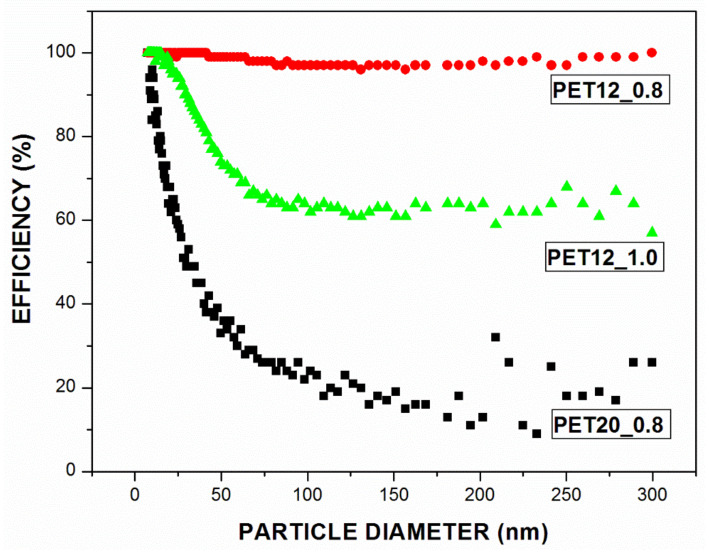
Fractional efficiency curves for samples with filtration velocity equal to 4.8 cm/s.

Therefore, knowing the concentration of the NaCl nanoparticles before and after passing through the filter media, it is possible to determine the overall and fractional efficiency of the nanoparticles of the filter media produced in this study. Figure 3 shows the efficiency curves as a function of the diameter of the nanoparticles. Sample PET12_0.8 (red curve) had a 98.4% overall efficiency, while samples PET12_1.0 (green curve) and PET20_0.8 (black curve) presented values of 76.8% and 38%, respectively (see Table 3). Due to the high fiber diameter obtained in PET20_0.8, the diffusion filtration mechanism characteristic of this nanoparticle size range was negatively affected reducing the global efficiency [36], as can be seen in Figure 3 which shows that, the efficiency decrease as the particle diameter increase.

For the PET12_1.0 sample (green curve), this decrease in efficiency with the nanoparticles diameter increase was less pronounced, more visible for nanoparticles diameter close to 70 nm. Yet, for PET12_0.8, the efficiency curve shows a slight drop for diameter particles close to 100 nm. This drop in the curve corresponds to the most penetrating particle size (MPPS) or regions of minimal efficiency, where for particles whose diameter is smaller than MPPS, diffusion mechanism is predominant and for particles with a larger size, the interception mechanism predominates. This occurs because during filtration, more than one collection mechanism is predominant, and in these regions, there is a greater particle penetration through the filter media [35]. 

When comparing all the samples, the higher global collection efficiency is due to the smaller diameter of the fibers of the sample, as also reported in the literature [68]. The variation in both pressure drop and global collection efficiency for the membranes studied is due to the large difference in the mean diameter of the fibers. For PET12_0.8 and PET12_1.0, samples with similar average diameters, the difference in filtration performance may be associated with the permeability of the filter media. As previously mentioned, from the results obtained for samples PET12_0.8 and PET12_1.0, it was possible to conclude that the increase in the feed rate from 0.8 mL/h to 1.0 mL/h caused an increase in the permeability constant, configuring the sample PET12_1.0 as a more permeable structure. Thus, a reduction in pressure drop was observed, which consequently led to less efficient collection, in this case.

In this work were studied microfiber filter media applied to nanoparticle filtration, thus, it can be inferred that the filter media produced had favorable characteristics for application in air filtration operations, such as high collection efficiency (98.4%) and low-pressure drop (212.5 Pa), as to the ones found in literature, where pressure drop values for HEPA filters are close to 269 Pa [32].

The electrospinning technique is widely used to produce filter media to control air pollutants, mainly aerosols [69,70,71]. In the research of Xia et al. (2018), they presented several data about pressure drop and face velocity of electrospun membranes for air filters [72]. For PAN nanofibers with similar face velocity, they found about 27 kPa of pressure drop in fibers with 330 nm of the average diameter. They also presented filtration efficiency for particles smaller than 300 nm for different electrospun filter media. Its efficiency was very high for most of the samples tested (more than 90%), but the face velocity was very low, which can restrict their application. 

The results found here indicate that the developed recycled PET electrospun microfiber can be used as an air filter media, and its performance is good. Due to the low-pressure drop found, the authors propose that it could be applied as indoor air filters in hospitals, ambulatories, clean zones of the pharmaceutical and food industry, aircraft, and bus cabins, among others. It can also improve the filtration performance in air conditioning equipment, gas–solid separation (such as bag filters), and face masks or respirators. This last proposed use is very interesting, mainly because of the recent waste generation caused by the coronavirus-2019 pandemic. It is mandatory to develop new clean and sustainable technologies to improve air quality and reduce the environmental impact, being better when the material is obtained from waste. 

## 4. Conclusions

In summary, the PET bottles waste filter media produced by the electrospinning technique proved to be excellent air filters to capture the nanoparticles and bioaerosols, with the high collection efficiency (98.4%) and low-pressure drop (212 Pa); in addition, the filter media had high mechanical resistance (4 MPa) and small-fiber diameters (1.29 µm). The study of the operational parameters influence was important to emphasize that the solution concentration variation is the factor that most affected the morphology of the fibers produced. It is noteworthy that the results obtained for both filter media, produced with flow rates of 0.8 and 1.0 mL/h and 12% of PET waste concentration, were very promising when applied to air filters, showing that they were highly efficient in removing dust smaller than PM2.5, especially for the dust super thin in the 100 nm size range, in which most viruses are found, such as the new coronavirus-19, present in the air. Consequently, this research will provide subsidies for future projects regarding the use of alternative materials to be used in the production of filter media for highly efficient air filters, mainly for indoor environments that need to be extremely clean and also for air conditioning equipment. Nowadays, due to the pandemic, it is of fundamental importance that air filters are efficient to collect nanoparticles and bioaerosols. The next study is to optimize the producing filter media process with electrospun PET bottle waste nanofibers, changing the variables, such as polymer concentration range, needle orifice size, collection time, among others, to further increase the filter media efficiency with the lowest pressure drop, reducing energy consumption spendings.

## Figures and Tables

**Table 1:** Operating parameters for electrospinning.

Samples	Polymer Concentration (wt%)	Flow Rates (mL/h)
**PET20_0.8**	20	0.8
**PET12_0.8**	12	0.8
**PET12_1.0**	12	1.0

**Table 2:** Characterization of PET fibrous filters.

Samples	Thickness (μm)	Average Fiber Diameter (μm)	Tensile Strength (MPa)	Porosity (Ergun Equation) (%)
PET20_0.8	392.50	3.25 (±1.86)	3.2	94 (±0.0004)
PET12_0.8	342.73	1.29 (±0.84)	3.5	92 (±0.0005)
PET12_1.0	365.12	1.27 (±0.76)	4.0	96 (±0.0038)

**Table 3:** Filtration performance of recycled PET microfiber filters.

Samples	Permeability Constant K_1_ (m^2^)	Pressure Drop (Pa) (*v_s_* = 4.8 cm/s)	Global Collection Efficiency (%)
PET20_0.8	2.2 × 10^−7^	13.5	38 (± 0.6)
PET12_0.8	1.07 × 10^−8^	212.5	98.4 (± 0.1)
PET12_1.0	4.4 × 10^−8^	64.8	76.8 (± 1.4)
